# Bicarbonate transport as regulator of antitumour immunity in pancreatic cancer

**DOI:** 10.1002/1878-0261.13400

**Published:** 2023-03-04

**Authors:** Emily J. Kay, Sara Zanivan

**Affiliations:** ^1^ Cancer Research UK Beatson Institute Glasgow UK; ^2^ School of Cancer Sciences University of Glasgow UK

**Keywords:** acidosis, cancer, immunotherapy, metabolism, T‐cells, tumour microenvironment

## Abstract

Bicarbonate transport is a pre‐existing mechanism of pH regulation in pancreatic ductal cells. In a recent study, Cappellesso *et al.* demonstrated that pancreatic ductal adenocarcinoma metabolic rewiring creates an acidic environment, enhanced by bicarbonate import into cancer cells via SLC4A4. This acidity favours protumourigenic immunosuppression. Targeting SLC4A4 neutralises environmental pH and restores antitumour immunity, sensitising tumours to immune checkpoint blockade.

## Graphical abstract

In a recent study, Cappellesso et al. targeted a pre‐existing mechanism of pH regulation via the bicarbonate transporter SLC4A4 in pancreatic ductal adenocarcinoma. This rewires cancer cell metabolism and reduces the acidity of the surrounding environment. Acidity supresses the ability of the immune system to target cancers. By targeting SLC4A4, antitumour immunity is restored, and tumours are sensitised to immunotherapy.

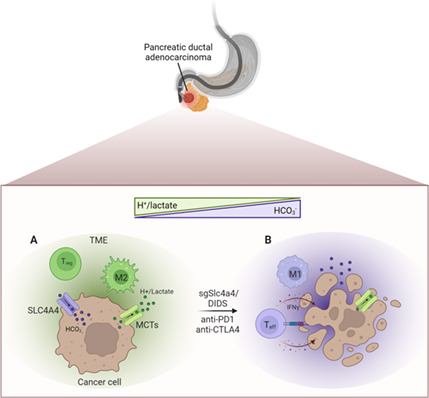

**Abbreviations**
ATP: adenosine triphosphate
CD69: cluster of differentiation 69
CTLA‐4: cytotoxic T‐lymphocyte associated protein 4
ICB: immune checkpoint blockade
IFNƴ: interferon gamma
KRAS: kirsten rat sarcoma viral proto‐oncogene
MCT‐1: monocarboxylate transporter 1
PD‐1: programmed cell death protein 1
PDAC: pancreatic adenocarcinoma
SLC4A4: solute carrier family 4 member 4
TME: tumour microenvironment

Treatment with immune checkpoint blockade (ICB) has revolutionised cancer treatment in the past decade and brought significant increases in patient survival with fewer side effects than standard chemotherapy. However, there are many solid tumour types where response to immunotherapy is minimal. Pancreatic adenocarcinoma (PDAC) is one such cancer. Pancreatic adenocarcinoma is characterised by a highly desmoplastic stroma. Pancreatic stellate cells, which are pancreas‐specific fibroblasts, are activated by PDAC to form a dense, collagen‐rich tissue, which acts as a barrier, blocking oxygen and nutrient delivery [1]. Therefore, one of the major metabolic reprogramming events in PDAC is increased glycolysis, commonly driven by oncogenic KRAS, to generate ATP and biomass under oxygen and nutrient stress [2]. Glycolysis leads to increased lactate secretion and a more acidic tumour microenvironment (TME), which favours the expansion of immunosuppressive T‐cell and myeloid cell subtypes (Fig. 1A). Furthermore, the stromal barrier prevents antitumour immune cell infiltration [1]. Attempts to deplete the stromal cells or collagen that comprise this barrier have proved ineffective in PDAC, enabling the aggressive PDAC tumour cells to expand and metastasise unhindered [3, 4]. However, depleting or targeting the stromal TME can also sensitise PDAC to ICB [5, 6]. Therefore, a more promising approach may be to reactivate the immune suppressed PDAC TME to improve antitumour immunity and response to immunotherapy. Cappellesso et al. [7] found a novel strategy for normalising the PDAC TME by targeting the cancer cells, specifically the bicarbonate transporter SLC4A4. This restores the balance between intracellular pH in cancer cells and the extracellular pH of the TME, thus enabling immune cell infiltration and antitumour response (Fig. 1B).

**Fig. 1:**
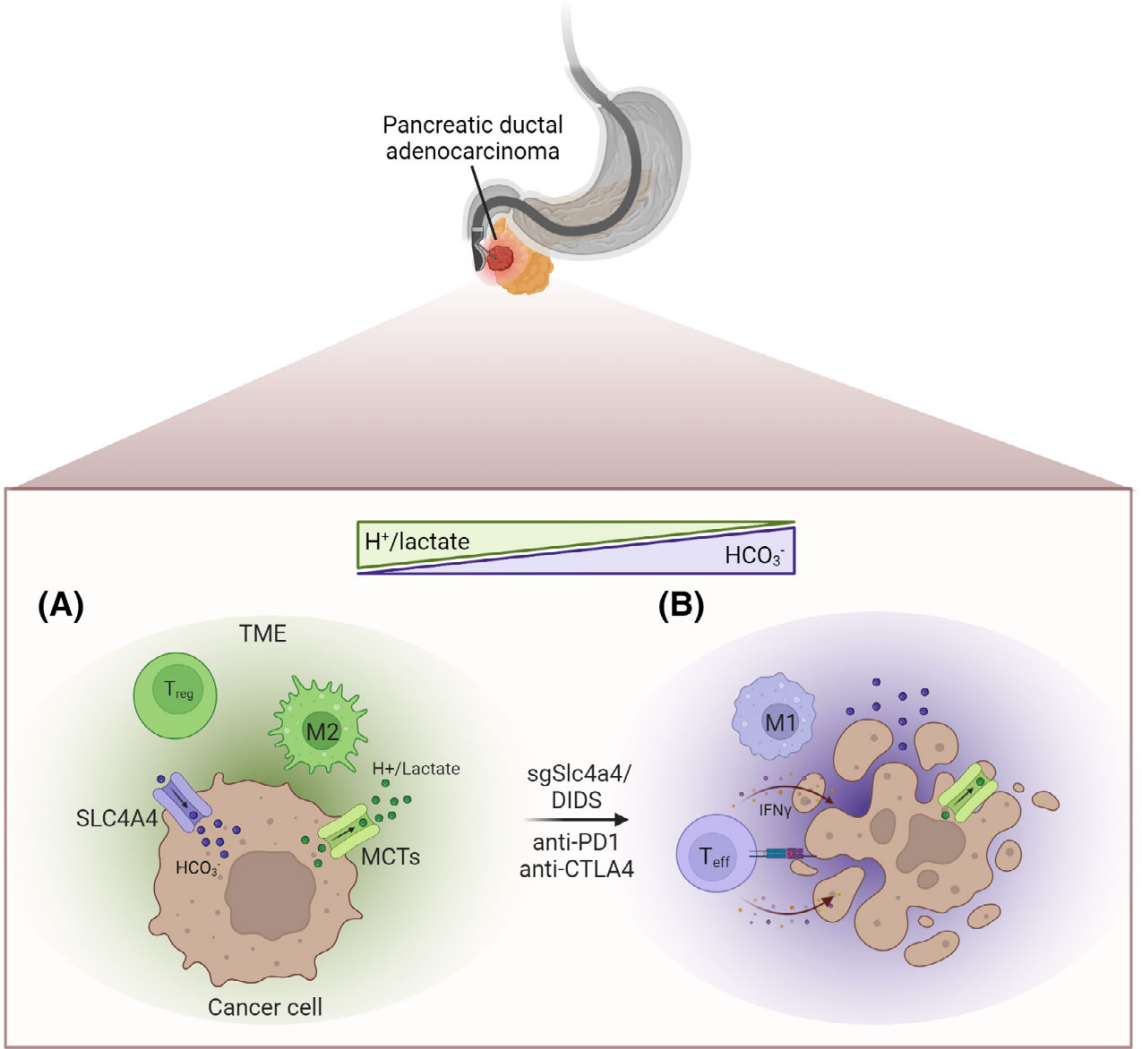
PDAC cancer cells retain the expression of SLC4A4, which imports bicarbonate (${\mathrm{HCO}}_{3}^{-}$), contributing to maintaining an acidic tumour microenvironment (TME) by increasing intracellular glycolysis and extrusion of protons (H^+^) and lactate via the monocarboxylate transporters (MCTs). Increased H^+^/lactate creates an immunosuppressive TME with Treg cells and M2‐like macrophages. Genetic depletion (sgSlc4a4) or inhibition of Slc4a4 with a non‐specific inhibitor 4,4′‐diisothiocyano‐2,2′‐stilbenedisulfonic acid (DIDS) reduces H^+^/lactate in the TME and restores CD8^+^ T‐cell infiltration and increased antitumour activation (M1‐like macrophages and IFNɣ expressing CD8^+^ T cells). Figure created with BioRender.com.

Bicarbonate transport is vital for normal pancreatic activity, as it needs to maintain an alkaline environment to control the activity of digestive enzymes secreted by pancreatic acinar cells. Normal ductal epithelial cells, which are thought to be the cells of origin for PDAC, secrete and import bicarbonate to maintain a pH that prevents the enzymes from self‐digesting the pancreas [8]. However, in PDAC, this balance becomes disrupted, and both the upregulation of glycolysis in cancer cells and the dense stromal barrier contribute to a highly hypoxic and acidic TME, on top of the continuing activity of bicarbonate import by ductal cells. It is well‐established that low pH and lactate levels create an immunosuppressive environment. Acidity suppresses the release of antitumour cytokines by T cells [9]. Furthermore, immunosuppressive Treg cells preferentially use lactate as a carbon source, and lactate polarises macrophages towards a more suppressive (M2) phenotype [10, 11]. However, targeting TME acidity has had mixed responses; while some targets, such as the monocarboxylate transporter MCT‐1, have made it to clinical trials, others still lack efficacious inhibitors [12, 13]. As yet, no one has successfully targeted the acidic TME in PDAC. The authors therefore identified bicarbonate importers as a potential target to restore pH levels in the PDAC TME. Of these, SLC4A4 was the most highly expressed in PDAC epithelial cells, according to their analysis of single‐cell RNA‐seq data from human PDAC tumours.

Interestingly, the authors showed that SLC4A4 is not actually upregulated in tumour cells compared with normal pancreatic epithelium. However, the presence of SLC4A4 in the restricted and hypoxic TME of PDAC could still fuel immunosuppressive acidity. Accordingly, Slc4a4 loss in murine PDAC cell lines increased extracellular pH (pH_e_) and lowered intracellular pH (pH_i_). A combination of tracing experiments with [^13^C]‐glucose, [^3^H]‐glucose and Seahorse metabolic analysis demonstrated that glycolysis and thereby lactate secretion was reduced in sgSlc4a4 knock‐out lines due to the inhibitory effect of low pH_i_ on glycolytic enzymes. Therefore, crucially, targeting Slc4a4 can not only increase pH_e_ via reducing bicarbonate import, but also via decreasing cancer cell lactate production, thus targeting both aspects of TME acidity that impair anticancer immunity.

The authors carried out an extensive *in vivo* characterisation of the effects of Slc4a4 targeting in orthotopic PDAC models. Two mouse PDAC lines were used for transplantation, Panc02 and KPC, both of which are resistant to ICB. The change in pH and decreased glycolysis caused by Slc4a4 loss *in vitro* was also demonstrated convincingly *in vivo*. Higher pH_e_ and lower pH_i_ in the tumour were detected using ^31^P magnetic resonance spectroscopy, and changes in glycolysis were measured by performing *in vivo* tracing experiments and calculating the ratio of ^13^C‐pyruvate to lactate conversion, as well as measuring the total levels of metabolites in the interstitial fluid. A reduction in tumour growth and metastasis to the lymph nodes and liver was observed in both models when Slc4a4 was deleted. The most dramatic effect was observed in the more immunogenic KPC_1_ clone, whereas the most aggressive and immune cold KPC_3_ clone was the least responsive, showing that highly immune cold tumours still present the greatest challenge for treatment. In support of this, the Panc02 and KPC_1_ tumours had increased CD8^+^ T‐cell infiltration and increased antitumour activation as measured by CD69 and IFNγ levels, whereas the KPC_3_ tumours did not show augmented CD8^+^ numbers but still had increased activation. Both Panc02 and KPC_1_ sgSlc4a4 tumours also had increased levels of M1‐like macrophages, which have antitumoural properties. Interestingly, this difference in macrophage activation was lost when the lines were transplanted into nude mice, leading to the novel observation that pH differences alone do not affect macrophage polarisation, but macrophages are in fact stimulated by pH‐dependent responses in the T cells, such as IFNγ production. Given that no difference in cancer cell proliferation was observed in the tumours, but rather increased cell death, this suggests that the immune response to pH normalisation is the main factor in reducing tumour aggressiveness in the PDAC models investigated in this study, rather than defects in the tumour cells caused by glycolysis inhibition.

Although targeting Slc4a4 boosted antitumour immune responses in the authors' models, a further question is whether this can sensitise PDAC to ICB therapy. Expression of the immune checkpoint markers PD‐1 and CTLA‐4 remained at the same level or higher in sgSlc4a4 tumours. Therefore, the authors chose to investigate whether the combination of anti‐PD‐1 and anti‐CTLA‐4 treatment enhanced the effect of sgSlc4a4 knockout. Unsurprisingly, the greatest response was observed in the KPC_1_ model, which was most responsive to the initial Slc4a4 deletion, and Panc02, which already showed a partial response to ICB in the sgNT control cell line. In both models, there was either complete regression or stable disease when ICB treatment was combined with sgSlc4a4 cell line transplantation. However, in the immune cold KPC_3_ model, Slc4a4 deletion still sensitised the tumours to ICB and reduced tumour growth even though no regression was observed. This suggested that the combination of SLC4A4 and ICB targeted therapy can still have value in more aggressive and less immunogenic tumours.

In summary, Cappellesso et al. have elegantly demonstrated two important findings for treatment of a highly aggressive and unresponsive cancer. First, that it is possible to effectively normalise the acidity of the PDAC TME by regulating bicarbonate transport, and that this reduces tumour aggressiveness. Second, that this sensitised tumours to ICB therapy, even in more immune cold and ICB‐resistant tumours. Therefore, SLC4A4 could be a valuable potential target for PDAC, which currently has few treatment options and where other attempts to target the TME have had limited success. For the future, an important step will be to develop specific SLC4A4 inhibitors that can be used for further validation. Although the authors used the SLC4A4 inhibitor 4,4′‐diisothiocyano‐2,2′‐stilbenedisulfonic acid (DIDS) with promising effects *in vivo*, the inhibitor is non‐specific and interacts with multiple anion exchangers [14]. It would also be useful to investigate whether SLC4A4 expression in PDAC patients correlates with immune cell infiltration, activation and response to immunotherapy, as this could indicate whether SLC4A4 regulates antitumour immunity in human patients and in mouse models.

## Conflict of interest

The authors declare no conflict of interest.
